# The efficacy and safety of fecal microbiota transplantation in the treatment of systemic sclerosis: A protocol for systematic review and meta analysis

**DOI:** 10.1097/MD.0000000000021267

**Published:** 2020-07-10

**Authors:** Shixiong Zhang, Jingjing Lv, Xuetong Ren, Xinyu Hao, Pingping Zhou, Yangang Wang

**Affiliations:** a Hebei University of Chinese Medicine.; b Hebei Province Hospital of Chinese Medicine, Shijiazhuang City, Hebei.

**Keywords:** fecal microbiota transplantation, protocol, systematic review, systemic sclerosis

## Abstract

**Background:**

Systemic sclerosis (SSc) is 1 of the most complex systemic autoimmune diseases.Accumulating evidence suggests that gut microbiota affect the development and function of the immune system and may play a role in the pathogenesis of autoimmune diseases. This new paradigm raises the possibility that many diseases result, at least partially, from microbiota-related dysfunction. This understanding invites the investigation of fecal microbiota transplantation (FMT) in the treatment of SSc. However, no study has specifically and systematically investigated the efficacy and safety of FMT in the treatment of SSc. Thus, this study will systematically and comprehensively appraise the efficacy and safety of FMT in the treatment of SSc.

**Methods:**

We will search the following sources without restrictions for date, language, or publication status: PubMed, Web of Science,Cochrane Central Register of Controlled Trials (CENTRAL) Cochrane Library, EMBASE and China National Knowledge Infrastructure. We will apply a combination of Medical Subject Heading (MeSH) and free-text terms incorporating database-specific controlled vocabularies and text words to implement search strategies. We will also search the ongoing trials registered in the World Health Organization's International Clinical Trials Registry Platform. Besides, the previous relevant reviews conducted on FMT for SSc and reference lists of included studies will also be searched.

**Results:**

This study will provide a reliable basis for the treatment of SSc with FMT.

**Conclusions:**

The findings will be an available reference to evaluate the efficacy and safety of FMT in the treatment of SSc.

**Registration number:**

INPLASY202060019.

## 1 Introduction

Systemic sclerosis (SSc), also known as scleroderma, is a rare connective tissue disease characterized by vascular and immune dysfunction, leading to fibrosis that can damage multiple organs.^[1–3]^ SSc is a clinically complex and challenging disease. It may be associated with high morbidity and mortality. A systematic review reported prevalence ranging from 3.8 per 100 000 in Taiwan to 50 per 100 000 in the USA. The prevalence was 23 per 100 000 (95% CI: 16–29 per 100 000; 18 studies) in a pooled sample of 11574 individuals. Incidence rate of SSc ranges from 0.77 per 100 000 person-years in the Netherlands to 5.6 per 100 000 person-years in the USA. SSc predominates in females with higher prevalence and incidence rates.^[4]^ Aside from this, it is a disease which may impact the quality of life. Also for the physician SSc is challenging. Due to its rarity, it is a disease which is uncommon to non-expert rheumatologists, which may lead to late diagnosis of the disease or its complications and jeopardisation of optimal management of the patient.^[5–8]^

SSc is 1 of the most complex systemic autoimmune diseases.^[9]^ The clinical and pathologic manifestations of the disease are the result of 3 distinct processes: innate and adaptive immune system abnormalities leading to production of autoantibodies and cell-mediated autoimmunity; microvascular endothelial cells and fibroproliferative vasculopathy of small vessels; fibroblast dysfunction leading to excessive collagen and other matrix components accumulation in skin, blood vessels, and internal organs.^[10–12]^ Despite the fact that in the last 20 years there has been improved understanding in the early diagnosis of the disease, and in identifying early internal organ involvement, therapy is still an unsolved problem.^[13,14]^ The heterogeneity in terms of extent, severity, and rate of progression of skin and internal organ involvement gives rise to many difficulties in finding the optimal therapeutic interventions for SSc and, to date, no disease-modifying agents are available.^[15]^

Accumulating evidence suggests that gut microbiota affect the development and function of the immune system and may play a role in the pathogenesis of autoimmune diseases.^[16–18]^This new paradigm raises the possibility that many diseases result, at least partially, from microbiota-related dysfunction.Specifically, patients with SSc have decreased abundance of beneficial commensal genera (eg, Faecalibacterium, Clostridium, and Bacteroides) and increased abundance of pathobiont genera (eg, Fusobacterium, Prevotella, Erwinia).^[19,20]^In addition, some studies have linked specific genera with the severity of gastrointestinal symptoms in SSc.^[21,22]^This understanding invites the investigation of fecal microbiota transplantation (FMT) in the treatment of SSc.

FMT is the transfer of stool from a healthy donor into the colon of a patient whose disease is a result of an altered microbiome, with the goal of restoring the normal microbiota and thus curing the disease.^[23]^FMT has been utilized sporadically for over 50 years. In the past few years, Clostridium difficile infection (CDI) epidemics in the USA and Europe have resulted in the increased use of FMT, given its high efficacy in eradicating CDI and associated symptoms. As more patients request treatment and more clinics incorporate FMT into their treatment repertoire, reports of applications outside of CDI are emerging, paving the way for the use of FMT in several idiopathic conditions, including SSc.^[24–26]^

Currently, there is no systematic review focusing on efficacy of FMT in the treatment of SSc with PCOS, so our meta-analysis aims to comprehensively explore it. Meanwhile we will provide high-quality evidence to help patients, clinicians as well as health policymakers select better treatment strategy of PCOS.

## 2 Objective

The objective of this systematic review is to identify, analyze and synthesize research evidence on the effectiveness and safety of FMT in the treatment of SSc.

## 3 Methods and analysis

### 3.1 Study registering and reporting

We have registered this study on INPLASY202060019. This systematic review protocol will be prepared to underlie the Preferred Reporting Items for Systematic Review and Meta-Analysis Protocols (PRISMA-P) guidance. We will record any protocol changes made during the implementation of the review in the publication of the final report. The PRISMA extension declaration is a declaration that ensures that all aspects of the method and result are reported. We followed the PRISMA-P guidelines.^[27]^

### 3.2 Eligibility criteria

The design of inclusion criteria and exclusion criteria in this study is based on the 5 main principles of PICOS.

#### 3.2.1 Type of Participants

We will include persons at least 18 years of age with a diagnosis of SSc (diffuse or limited type) as defined by the trial authors, with cutaneous or pulmonary involvement, or both. It will not be necessary that persons fulfil the preliminary American College of Rheumatology (ACR) criteria for scleroderma (ACR 1980); or 2013 ACR/EULAR Classification Criteria for Scleroderma (van den Hoogen 2013). Trials can include persons with any subset of scleroderma.

#### 3.2.2 Type of Interventions and comparators

Our intervention is FMT.And our comparator will be another standard of care for SSc treatment or nonexposure when applicable depending on the study.

#### 3.2.3 Outcomes

The Main outcomes are the fficacy and safety of FMT in the treatment of SSc. And the additional outcomes included:

(1) Percent predicted total lung capacity (TLC)
(2) Renal function - measured by creatinine clearance, estimated glomerular filtration rate (eGFR), and serum creatinine (percent of persons with a creatinine above normal).
(3) Cardiac function - measured by echocardiogram (to detect changes in ejection fraction).
(4) Health-related quality of life (HRQOL) - including pain measured by a visual analogue scale (VAS) and the Medical Outcome Survey Short Form (SF-36).
(5) Safety outcomes - withdrawals from study and adverse events reported as defined by the authors.
(6) Inflammatory markers - measured by erythrosedimentation rate (ESR) or C-reactve protein (CRP), or both.

#### 3.2.4 Study design

In order to limit heterogeneity and enhance clinical applicability, strict inclusion/exclusion criteria were established. Only the randomized controlled trials were included for analysis. We will rule out repeated studies that do not have enough information to calculate effect estimates. We will not apply any language or other restrictions.

### 3.3 Information source

We will search the following sources without restrictions for date, language, or publication status: PubMed, Web of Science,Cochrane Central Register of Controlled Trials (CENTRAL) Cochrane Library, EMBASE and China National Knowledge Infrastructure. We will apply a combination of Medical Subject Heading (MeSH) and free-text terms incorporating database-specific controlled vocabularies and text words to implement search strategies. We will also search the ongoing trials registered in the World Health Organization's International Clinical Trials Registry Platform. Besides, the previous relevant reviews conducted on FMT for SSc and reference lists of included studies will also be searched.

### 3.4 Search strategy

Two authors will screen the titles and abstracts of the all records retrieved in above electronic databases independently to find potentially eligible reviews. According to the inclusion and exclusion criteria outlined above, the full texts of them will be retrieved for further identification. Any disagreement will be resolved by discussion or by consultation with a third author. The search strategy for PubMed is presented in Table 1 and the strategy will be modified upon the requirement of other databases.

**Table 1:**
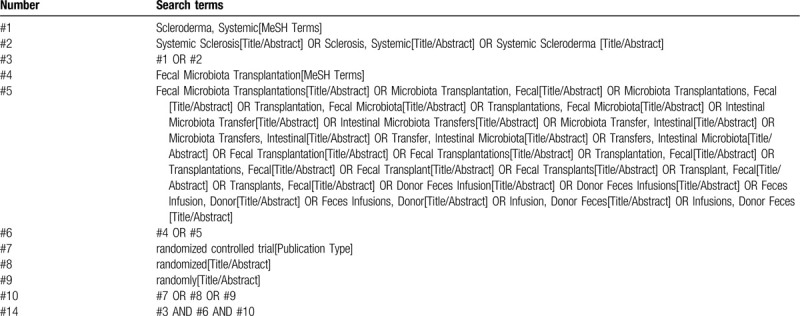
Search strategy used in PubMed database.

### 3.5 Data collection and analysis

#### 3.5.1 Study selection

Two reviewers will perform literature screening, study selection, and data extraction independently. The literature obtained will be imported into EndnoteX9 to screen the title and abstract, the duplications and studies failing to meet the pre-specified inclusion criteria will be excluded. After reading the full text of the remained literature and discussing within the group, the final included studies will be determined. The corresponding author of original RCT will be contacted when the full text is unavailable. Disagreements will be solved by consulting a third-party arbitrator or discussing within a group.

#### 3.5.2 Data extraction and management

Two authors will screen the titles and abstracts of the all records retrieved in above electronic databases independently to find potentially eligible reviews. According to the inclusion and exclusion criteria outlined above, the full texts of them will be retrieved for further identification. Any disagreement will be resolved by discussion or by consultation with a third author.

Data will be extracted by 2 reviewers independently using a pre-designed data extraction form. A third reviewer will validate data. The following data will be extracted: General information, Trial characteristics, Intervention(s) and control(s), Participants, Study methodology, Outcomes, Results, and so on.

#### 3.5.3 Risk of bias in included studies

The methodological quality of eligible studies will be assessed by 2 review authors independently according to the the Cochrane Handbook for Systematic Reviews of Interventions. The following characteristics will be assessed: random sequence generation (selection bias), allocation concealment (selection bias), blinding of participants and personnel (performance bias), blinding of outcome assessment (detection bias), incomplete outcome data (attrition bias), selective reporting (reporting bias), other bias. Based on the assessments of the studies against these seven domains, they will be classified as being of “low risk”, “high risk” or “unclear risk” of bias. Any disagreements will be resolved by discussion or discussed with another reviewer if necessary.

#### 3.5.4 Strategy of data synthesis

Meta-analysis was conducted using Review Manager software (version 5.3). Odds ratio (OR) with 95% confidence intervals was reported for the dichotomous data, and mean differences (MD) with 95% confidence intervals for the continuous data. Statistical heterogeneity between studies was tested by calculating Higgins *I*^2^ values or using the *χ*^2^ test. *I*^2^ > 25%, *I*^2^ > 50%, and *I*^2^ > 75% were respectively defined to indicate moderate,substantial, and considerable heterogeneity. When the *P*-value of *χ*^2^ test was < .1, an *I*^2^ test was carried out. If the *I*^2^ test showed a value >50%, a random effects model was carried out. Otherwise, a fixed effects model was carried out. A *P* value lower than .05 was considered to be statistically significant.

#### 3.5.5 Subgroup analysis

If results of the meta analysis are significantly heterogeneous,subgroup analyses of the control groups might be performed.

#### 3.5.6 Sensibility analysis

If sufficient trials are identified, we plan to conduct a sensitivity analysis comparing the results using all trials with high methodological quality: studies classified as having a ‘low risk of bias’ versus those identified as having a ‘high risk of bias’.

#### 3.5.7 Patient and public involvement

This is a meta-analysis study based on previously published data, so patient and public involvement will not be included in this study.

#### 3.5.8 Grading the quality of evidence

The Grading of Recommendations Assessment, Development and Evaluation guidelines will be utilized to grade the quality of evidence as very low, low, moderate, or high.

## 4 Discussion

SSc is an autoimmune disorder of unknown etiology characterized.^[28]^It affects the gastrointestinal tract in more than 80% of patients.Any part of the gastrointestinal tract can be affected, from the mouth to the anus.^[29]^ Recently, the pathogenesis of SSc has been reported to be related to the dysbiosis of gut microbiota.^[30,31]^Though there are several issues to be understood, FMT has been as an innovative approach to SSc.^[32]^However, as far as we know,no literature review systematically assesses the efficacy and safety of FMT in the treatment of SSc. Therefore, this systematic review will investigate the efficacy and safety FMT in the treatment of SSc. We expect that this study may provide a basis for FMT for the treatment of SSc, and may provide better options for the treatments to such patients.

## Author contributions

**Conceptualization:** Yangang Wang.

**Data curation:** Shixiong Zhang.

**Formal analysis:** Jingjing Lv.

**Funding acquisition:** Yangang Wang.

**Methodology:** Xuetong Ren.

**Software:** Xinyu Hao, Pingping Zhou.

**Supervision:** Yangang Wang.

**Writing – original draft:** Shixiong Zhang.
